# Hydrodynamic characterization of a vesicular stomatitis virus-based oncolytic virus using analytical ultracentrifugation

**DOI:** 10.1007/s00249-023-01649-w

**Published:** 2023-05-03

**Authors:** Simon Wawra, Sophia Kessler, Arina Egel, Johannes Solzin, Oliver Burkert, Daniel Hochdorfer

**Affiliations:** 1 Boehringer Ingelheim Pharma GmbH & Co. KG, Innovation Unit, Analytical Development Biologicals, Biberach, Germany; 2 Boehringer Ingelheim Pharma GmbH & Co. KG, Innovation Unit, Viral Therapeutics Center, Biberach, Germany; 3 Boehringer Ingelheim Therapeutics GmbH, Innovation Unit, Viral Therapeutics Center, Ochsenhausen, Germany

**Keywords:** Analytical ultracentrifugation, Vesicular stomatitis virus, Oncolytic virus, Nanoparticle tracking analysis

## Abstract

Determination of the size, density, and mass of viral particles can provide valuable information to support process and formulation studies in clinical development. Analytical ultracentrifugation (AUC), as a first principal method, has been shown to be a beneficial tool for the characterization of the non-enveloped adeno associated virus (AAV). Here, we demonstrate the suitability of AUC for the challenging characterization of a representative for enveloped viruses, which usually are expected to exhibit higher dispersity than non-enveloped viruses. Specifically, the vesicular stomatitis virus (VSV)-based oncolytic virus VSV-GP was used to evaluate potential occurrence of non-ideal sedimentation by testing different rotor speeds and loading concentrations. The partial specific volume was determined via density gradients and density contrast experiments. Additionally, nanoparticle tracking analysis (NTA) was used to determine the hydrodynamic diameter of VSV-GP particles to calculate their molecular weight via the Svedberg equation. Overall, this study demonstrates the applicability of AUC and NTA for the characterization of size, density, and molar mass of an enveloped virus, namely VSV-GP.

## Introduction

A fast-growing number of virus-based therapeutics are entering clinical development, such as adeno-associated virus (AAV) for gene therapy and vesicular stomatitis virus (VSV) for both oncolytic cancer therapy (Muik et al. 2014; Merchan et al. 2020) and modern vaccination approaches (Piszczatoski and Gums 2020; Saphire 2020; Liu et al. 2021). During development, analytical characterization methods are essential to lead process and product design of viral therapeutics. In particular, development activities benefit from analytical techniques that enable the characterization of size, density, molar mass, and aggregation of virus particles with high accuracy. This information can then be used for the assessment of critical quality attributes, e.g., the ratio of full to empty particles for AAV. For AAV, analytical ultracentrifugation (AUC) has already been extensively used and has been shown to be a valuable tool for characterization of size and composition heterogeneity (Berkowitz and Philo 2007; Burnham et al. 2015; Fu et al. 2019; Maruno et al. 2021) and probably can be considered the gold standard for quantification of empty and full capsids (Gimpel et al. 2021). In contrast, less AUC data on other viral therapeutics like VSV-GP (Muik et al. 2014) are available. VSV is a member of the Rhabdoviridae and is known for its bullet-shaped morphology. VSV has an approximate size of 175 nm × 70 nm (David-West and Labzoffsky 1968), molecular weights were reported between 265 and 355 MDa (Ware et al. 1973; Hartford et al. 1975; Thomas et al. 1985), and sedimentation coefficients between 610 and 667S (Bradish et al. 1956; Ware et al. 1973; Hartford et al. 1975). As VSV is an enveloped virus, a higher degree of polydispersity is expected compared to the non-enveloped AAV, because the envelope is less well defined regarding its composition compared to the protein-based capsid. In addition, it has been reported that nucleocapsids are not homogenous with regards to their radial diameter, which consequently leads to a polydisperse nucleocapsid length distribution (Desfosses et al. 2013; Jenni et al. 2022). In this contribution, we show an experimental approach for the development of analytical ultracentrifugation methods for characterization of VSV-GP. We determined dispersed sedimentation, density, spectral, and diffusion properties leading to the assessment of the molecular weight of VSV-GP. This approach has great potential for other enveloped viruses of similar size, e.g., lentiviruses, and lipid nanoparticles.

### Experimental background

#### Analytical ultracentrifugation

Although the basis of the various existing experimental techniques can be found in literature (Schuck et al. 2015; Uchiyama et al. 2016), the main principles relevant for this study will be outlined in the following.

The sedimentation velocity of particles and macromolecules within a centrifugal field $\dot{r}$ can be normalized to the applied centrifugal field with angular rotor velocity $\omega$ and radial position $r$ giving the sedimentation coefficient $s$, which solely depends on particle and solvent parameters, such as, the mass of the particle $m$, the solvent density ${\rho }_{S}$, the friction factor $f$, as well as the particle’s partial specific volume $\overline{v }$, which is related to the inverse of the particle density.

1 $$s=\frac{\dot{r}}{{\omega }^{2}r}=\frac{m(1-\overline{v}\cdot {\rho }_{S})}{f}$$

Radial broadening of the sedimentation boundary during the experiment can be attributed to sample polydispersity in terms of the sedimentation coefficient or can be due to the influence of diffusion, which relates to the diffusion coefficient $D$ with the thermal energy ${k}_{B}T$:

2 $$D=\frac{{k}_{B}T}{f}$$

The frictional factor is given based on the hydrodynamic diameter ${x}_{H}$ and the solvent viscosity $\eta$:

3 $$f=3\pi \eta {x}_{H}$$

An alternative to AUC for obtaining the diffusion coefficient of particles is by tracking the Brownian motion of particles over time via, e.g., nanoparticle tracking analysis (NTA). Once D and s are known, the Svedberg equation can be used to determine the particle’s molar mass $M$ via the Avogadro constant ${N}_{A}$ (Svedberg 1925):

4 $$M=\frac{s}{D}\frac{{k}_{B}{N}_{A}T}{\left(1-\overline{v}\cdot {\rho }_{S}\right)}$$

Overall, the sedimentation coefficient and the diffusion coefficient indicate the potential of analytical ultracentrifugation to provide insights into hydrodynamic, thermodynamic and density properties of virus particles depending on the experimental approach.

Another type of experiment, which would rather be considered a sedimentation equilibrium experiment (Meselson et al. 1957), relies on the occurrence of a static concentration gradient, e.g., made of CsCl, leading to a radially varying density of the solvent, thus, providing the possibility of detecting buoyant density heterogeneity (Berkowitz and Philo 2007). This density gradient method (Mächtle and Börger 2006) forces the particles to sediment and float to the isopycnic position, where according to Eq. 1 the sedimentation coefficient becomes zero, hence particles neither float nor sediment. Broadening of detected bands can then be attributed either to buoyant density dispersity or diffusion of the particles, which can be tuned, e.g., via the steepness of the density gradient (Vinograd 1963). The radial static gradient can be either determined via tracer particles or modeled based on meniscus and bottom position of the cell used with ${\varphi }_{1}^{in}$ being the initial volume fraction of CsCl and ${\varphi }_{0}^{in}$ the initial volume fraction of water (Mächtle and Börger 2006):

$${\rho }_{s}\left(r\right)={\rho }_{0} + {\rho }_{1}\cdot \alpha \cdot \mathit{exp}\left(\beta \cdot {r}^{2}\right)$$

$$\alpha =\frac{\mathit{exp}\left(\beta \cdot {\varphi }_{1}^{in} \cdot \left({r}_{b}^{2} - {r}_{m}^{2}\right)\right) - 1}{\mathit{exp}\left(\beta \cdot {r}_{b}^{2}\right)-\mathit{exp}\left(\beta \cdot {\varphi }_{1}^{in} \cdot {r}_{b}^{2} + \beta \cdot {\varphi }_{0}^{in} \cdot {r}_{m}^{2}\right)}$$

5 $$\beta = \frac{{\omega }^{2} \cdot \left(\frac{{M}_{1}}{{\rho }_{1}}\right) \left({\rho }_{1} - {\rho }_{0}\right)}{2RT}$$

Another method targeting the partial specific volume of the particles or macromolecules in solution, relies on the partial exchange of solvent via isotope exchange, thereby, altering viscosity and density of the solvent (Cheng and Schachman 1955; Martin et al. 1956; Mächtle 1984). There, influencing factors might be a partial H–D exchange (Brown et al. 2011; Fagan et al. 2013; Henrickson et al. 2021; Maruno et al. 2021) or the incorporation of density varying components into the investigated particles. From the cumulative sedimentation coefficient distributions measured at several different isotope levels, the change of sedimentation coefficient for the individual distribution fraction can be used alongside the solvent density to deduce the apparent partial specific volume ${\overline{v} }_{app}$ via linear extrapolation to the balance of centrifugal and buoyancy force:

6 $${\overline{v} }_{app}=\frac{1}{{\rho }_{S}(s\cdot \eta =0)}$$

The data obtained should be checked for consistency, as the analysis can be misled by substantial density heterogeneity (Mächtle 1984). Therefore, a prerequisite is that the order of species within the sedimentation boundary, respectively, the sedimentation coefficient distribution, should not alter to avoid incorrect attribution of partial specific volumes per fraction.

#### Nanoparticle tracking analysis

In NTA, particles are detected by light scattering and the Brownian motion of individual particles is measured in a video. The two-dimensional displacement ($<{\overline{\mathrm{x },\mathrm{y}}}^{2}>$) of the particles over time $\mathrm{t}$ is used to assess the diffusion of the particles.

7 $$D = \frac{{ < \overline{{x,y}} ^{2} > }}{{4 \cdot t}}$$

Using the Stokes–Einstein equation, the hydrodynamic diameter of the samples can then be calculated.

## Materials and methods

### Analytical ultracentrifugation

AUC experiments were performed with a Beckman Coulter Optima AUC using absorbance optics. For all experiments an An50-Ti rotor was used, and experiments were run at 20 °C.

### Sedimentation velocity experiments (SV)

SV experiments were performed using epon double-sector centerpieces, which were filled with 450 µL of sample. Samples were diluted in their corresponding formulation buffer. Scans were obtained at 280 nm and 320 nm. The samples were measured at 2000–12,000 rpm. Analysis of absorbance and pseudo-extinction data was performed using Sedfit (Schuck and Rossmanith 2000) (resolution of 100, no regularization, fitting of RI-, TI-noise and meniscus). Density and Viscosity of formulation buffers were measured using a DMA density meter and a Lovis viscosimeter from Anton Paar.

### Density gradient experiments

Density gradient experiments were performed with epon double-sector centerpieces, which were filled with 450 µL of sample in formulation buffer spiked with 17 and 15 wt% cesium chloride for material A and B, and stressed material C, respectively. The samples were centrifuged at 35,000 rpm for a total of 34 h, scanning at 280 nm every 30 min for the first 26 h and then scanning every other wavelength between 240 and 320 nm. Afterwards, this was repeated at 40,000 rpm. The particle buoyant density was calculated for the CsCl using Eq. 5 based on the meniscus and bottom radial positions determined in the first scan. The initial CsCl concentration in the sample compartment was calculated based on the spiked CsCl fraction. A constant density increment was added to the radial density distribution of CsCl to account for remaining formulation components.

### Density contrast experiments

H_2_O/D_2_O Experiments were performed with epon double-sector centerpieces, which were filled with 450 µL of sample diluted in formulation buffer with varying concentrations of D_2_O (0%, 25%, 50%). Density and viscosity were calculated using SEDNTERP (Philo 2021) taking the buffer composition into account. The experiment was run at 6000 rpm and scans were acquired at 280 nm. Sedimentation coefficient distributions were obtained with Sedfit using the ls-g(s*) model and the distribution of ${\overline{v} }_{app}$ was extrapolated.

### Viruses

VSV, in which the glycoprotein (G) of VSV was replaced by the glycoprotein (GP) of lymphocytic choriomeningitis virus, was used (Muik et al. 2014). Material A, B, and C are from pre-clinical process development. Material C has additionally been subjected to surfactant stress prior to characterization.

### Nanoparticle tracking analysis

For nanoparticle tracking analysis (NTA) the ZetaView^®^ PMX-120 from Particle Metrix was used. Samples were diluted in their corresponding formulation buffer and viscosity of the buffer (calculated in SEDNTERP (Philo 2021)) was taken into consideration for determination of the hydrodynamic diameter. Three replicates of each sample at 11 positions with 5 cycles each were measured.

## Results and discussion

### Evaluation of non-ideality and optimization of SV-experiment

The wildtype of VSV was reported to be anisotropic (approximately 175 × 70 nm (David-West and Labzoffsky 1968)) giving an aspect ratio of approximately 2.5 and a sedimentation coefficient between 610 and 667S (Bradish et al. 1956; Ware et al. 1973; Thomas et al. 1985). The influence of the concentration and rotor speed on the retrieved sedimentation coefficients for anisotropic particles has been discussed and investigated extensively in literature for various macromolecules like DNA, the tobacco mosaic virus, or carbon nanotubes (Hearst and Vinograd 1961; Batista et al. 2014; Schuck et al. 2015). Therefore, the influence of rotor speed and concentration on the retrieval of sedimentation coefficient distributions as well as the retrieved subspecies of VSV-GP were investigated using a stressed sample (material C) and two different unstressed samples (material A and B). In accordance to protein analysis nomenclature, we named the subspecies fragment, main and oligomer fraction, however, the required analysis for a final characterization and identification goes beyond the scope of this work.

**Fig. 1:**
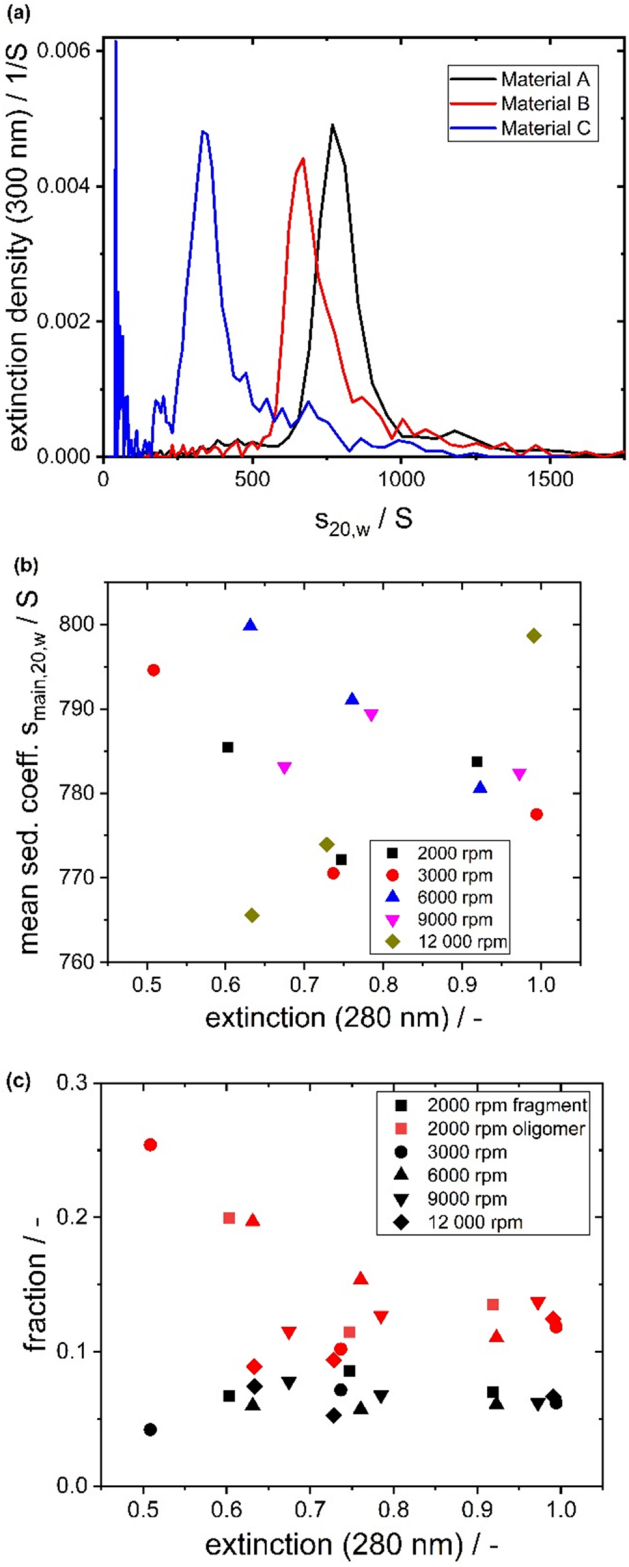
Results of the AUC characterization of material A, B, C. **a** Sedimentation coefficient distributions at 6000 rpm transformed to water conditions based on ${\overline{\mathrm{v}} }_{ }=0.842\frac{{\mathrm{cm}}^{3}}{\mathrm{g}}$ **b** Mean s-value from peak integration between 620 and 980S for material A depending on loading extinction signal. **c** Integrated relative fraction of fragments and oligomers in remaining parts of distribution for material A depending on loading extinction signal. Symbols indicate rotor speed, the colors give information on the type of subspecies namely oligomer or fragments

The results of these studies are depicted in Fig. 1, where the main species of material A exhibits a modal value of about 769S at 20 °C and water conditions. Varying the rotor speed and loading concentration did not significantly influence the retrieved sedimentation properties. These results suggest that non-ideal sedimentation is not relevant within the experimental range used here. Apart from the main species, the exemplary sedimentation coefficient distribution of material A shows a slower sedimenting species below 620S and a species with a higher sedimentation coefficient. To evaluate whether the faster sedimenting species is in accordance with the sedimentation speed of a virus particle dimer, theoretical estimations using the Zeno software can be performed (Juba et al. 2017). There, the hydrodynamic properties of dimers made of spherical, cuboid or rod-like particles can be evaluated quickly. Using those geometrical models, the sedimentation coefficients of dimers can be estimated to be between 900 and 1500S, considering the sedimentation coefficient distribution width of the main species. It is obvious that due to the virus’ inherent polydispersity in terms of size, envelope composition, density and shape, the analysis and assignment of oligomers is more challenging than for other biopharmaceuticals, such as antibodies (Bou-Assaf et al. 2022). However, the amount of oligomers can be an important quality attribute during development of a virus-based therapeutic, as oligomers and larger aggregates might lead to potency loss and trigger immunotoxicity (Wright 2014; Gimpel et al. 2021). Therefore, the relative absorption-based amount is an important parameter, especially during process optimization. The results of the data evaluation in Fig. 1 were not showing a significant influence of rotor speed or initial concentration for oligomers or fragments. However, the determined relative amount of the faster sedimenting species showed larger variations, which could be caused by the less pronounced separation between main and oligomeric species. Overall, the results of these experiments suggest that rotor speed and concentration do not influence the retrieved sedimentation properties to great extent, allowing the use of higher rotor speeds and thus achieving higher sample throughput without compromising the data quality.

For material B, the sedimentation coefficient distribution in Fig. 1 exhibits a similar shape but exhibits minor amounts of faster sedimenting subspecies above 2500S (not shown). Albeit significant overlap with the sedimentation coefficient distribution of material A, the corresponding modal value of material B is slightly shifted to smaller sedimentation coefficient values (671S), which might be due to small differences in density, mass or geometry of the viral particle according to the definition of the sedimentation coefficient in Eq. 1. The exhibited differences in sedimentation properties of material A and B show the ability of analytical ultracentrifugation to provide additional information on product quality supporting the expectation, which were derived from the analytical panel of orthogonal methods performed in-house (data not shown).

Lastly, material C, which was subjected to chemical stress, shows significantly smaller sedimentation velocities, as shown by a modal value of 332S. According to the definition of the sedimentation coefficient in Eq. 1, this reduced sedimentation velocity could be attributed to a reduction of density or mass, or the combination of those factors. To get more insights into the differences of these disperse enveloped viral particles, additional experiments targeting those quantities are needed.

### Density determining experiments

**Fig. 2:**
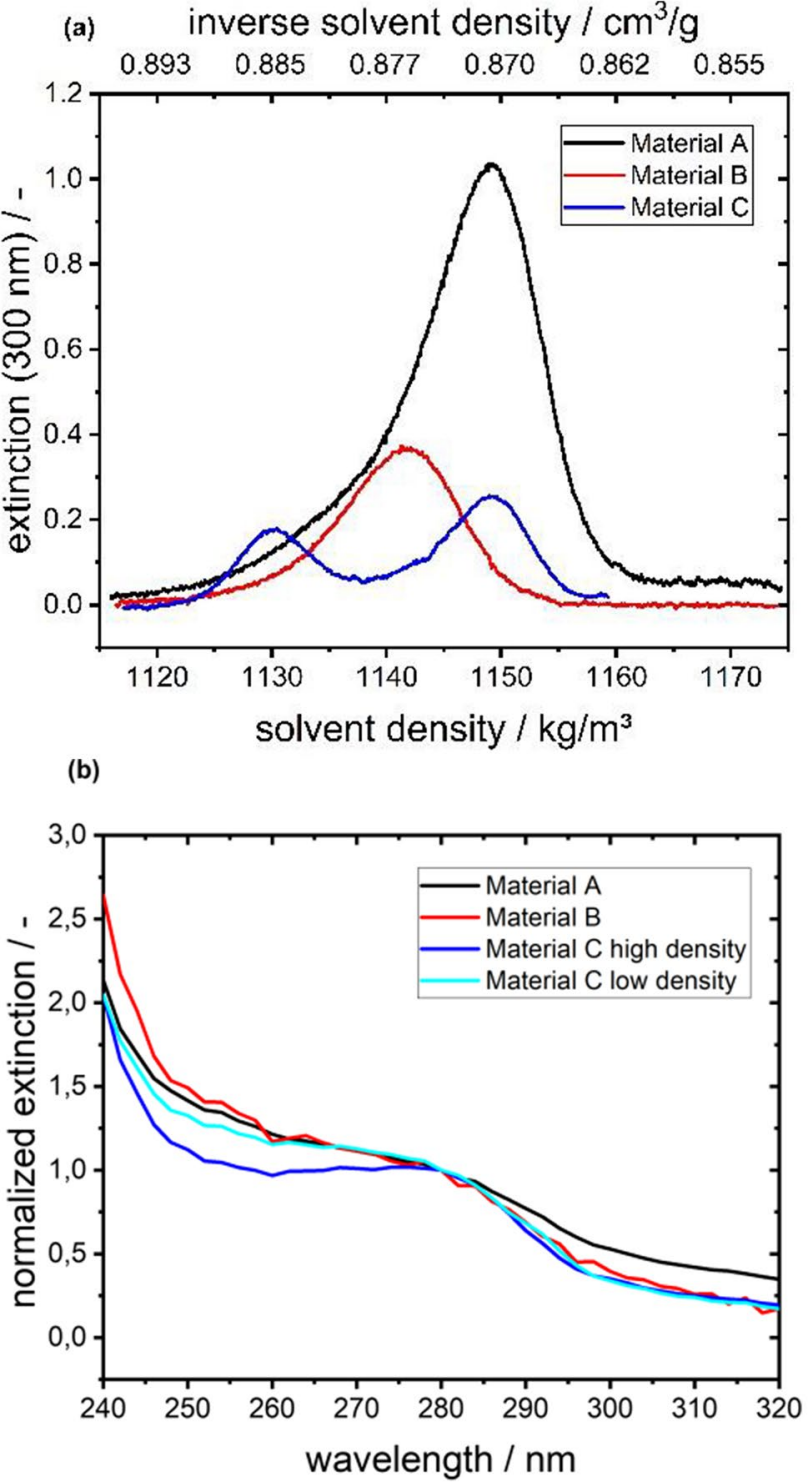
Results of density gradients experiments using CsCl. **a** Radial distribution of extinction **b** Extracted extinction spectra of subspecies normalized to 280 nm

Characterizing the density heterogeneity of a formulated viral vector can provide valuable information on critical quality attributes like the ratio of empty to full AAV vectors (Berkowitz and Philo 2007), or can be used for batch-to-batch comparisons. In this study, we used CsCl density gradients and H_2_O/D_2_O-density variation to get access to buoyant density information of the sedimenting particles. The results in Fig. 2 showed two occurring subspecies in material C, while material A and B only exhibited one species. Compared to density gradient data from literature for wildtype VSV (McCombs et al. 1966) with species in CsCl at solution densities of 1190, 1220 and 1260 kg/m^3^ and one species in sucrose at 1160 kg/m^3^, we found for material A and B only one VSV-GP species with a smaller density ranging from 1140 to 1150 kg/m^3^ and two species for material C with 1130 and 1150 kg/m^3^. Apart from the composition difference of VSV and VSV-GP, which may lead to different density, the stability of the virus particles during the density gradient experiment might be an issue. McCombs et al*.* argue that VSV disruption via CsCl might occur and could lead to high density bands, whereas no disruption in sucrose occurs, and therefore, results in a single band only. The bands we observed stayed at the same position throughout the experiment for 34 h at 35,000 rpm and 34 h at 40,000 rpm, therefore, we assumed that the material was not disrupting the virus particles on the timescale of the experiment.

With the radial separation of the components, measurement of extinction spectra of the individual subspecies with high spectral resolution via the Optima AUC was practicable. This revealed that material C is not only consisting of two species with varying density, but also that these species have differing spectra, as the normalized spectra in Fig. 2 diverge in the region of 240 nm to 280 nm. In particular, the two species differed in their 260 nm to 280 nm ratio. An explanation for this could be that the induced stress led to loss of the viral genome. Surprisingly, the species with the lower 260 nm to 280 nm ratio is also the species with the higher density. Further experiments involving cryo-EM to investigate the nature of these subspecies as well as the determination of the molar mass could help resolving this open question. However, the latter is challenging to achieve, as the sedimentation coefficient distribution of material C in Fig. 1 does not exhibit two distinct species.

**Fig. 3:**
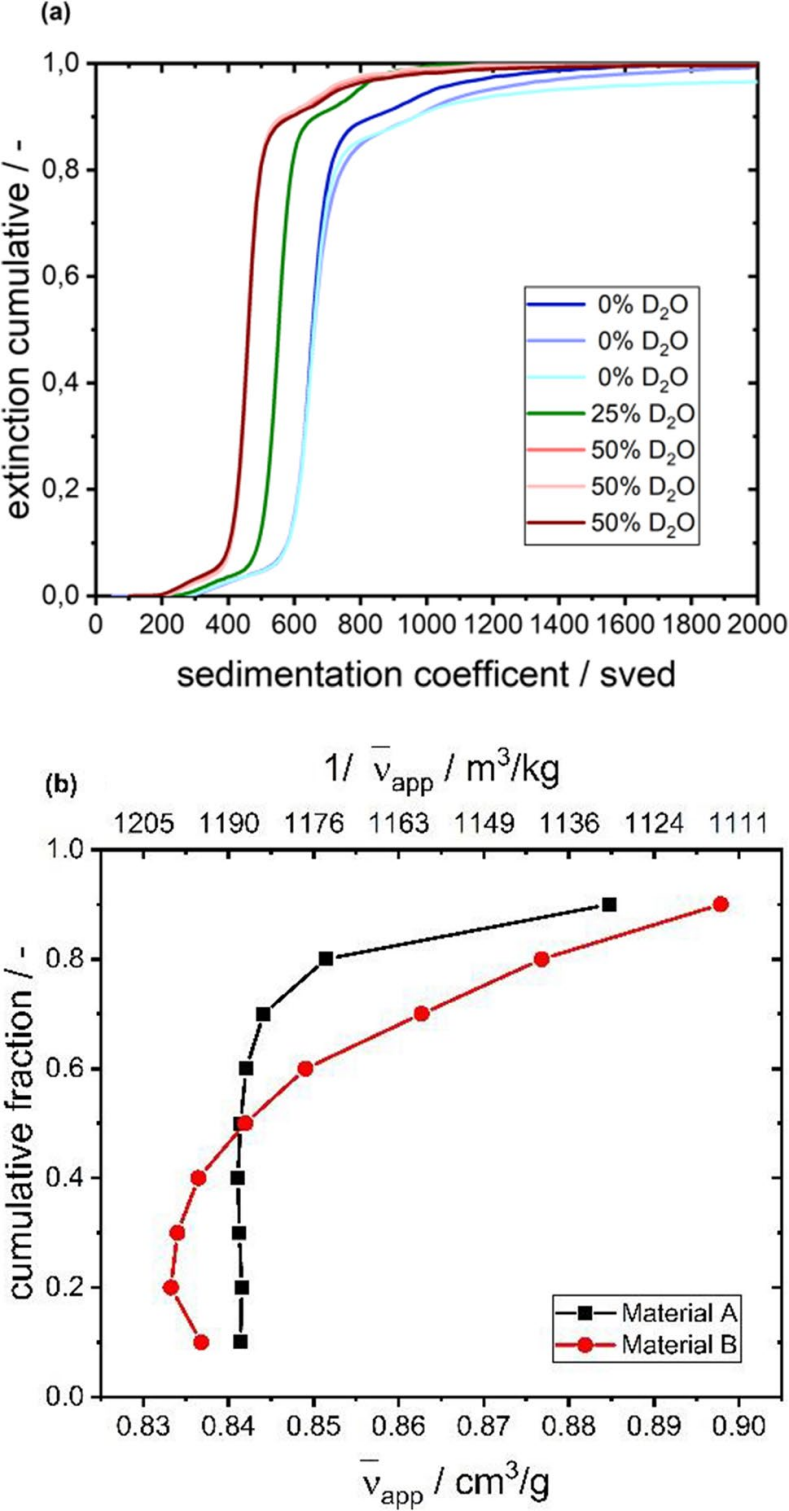
Results of density variation via H_2_O/D_2_O **a** Cumulative sedimentation coefficient distribution for material A at different D_2_O-concentrations **b** resulting  ${\overline{\mathrm{v}} }_{\mathrm{app}}$ for cumulative fractions in (**a**)

To avoid any uncertainty in terms of sample stability, heavy water can be used to vary the density of the solution (Cheng and Schachman 1955; Martin et al. 1956; Mächtle and Börger 2006). The original sedimentation coefficient distributions shown in Fig. 3, suggest that especially for low and high boundary fractions the calculation of the partial specific volume is difficult. This is due to the fact that the slowly varying cumulative sedimentation coefficient distributions in the low or high fractions might not be well defined experimentally, which could lead to these alterations of individual data points. Therefore, these parts are omitted from the analysis.

Compared to the results from the CsCl density gradient experiments, the measured partial specific volumes in H_2_O/D_2_O were a few percent lower. This could be attributed to membrane penetration of D_2_O (Fettiplace and Haydon 1980) or H–D exchange (Brown et al. 2011; Fagan et al. 2013; Henrickson et al. 2021; Maruno et al. 2021), which could lead to an overestimation of the measured density in this experiment.

### Determination of molar mass

**Fig. 4:**
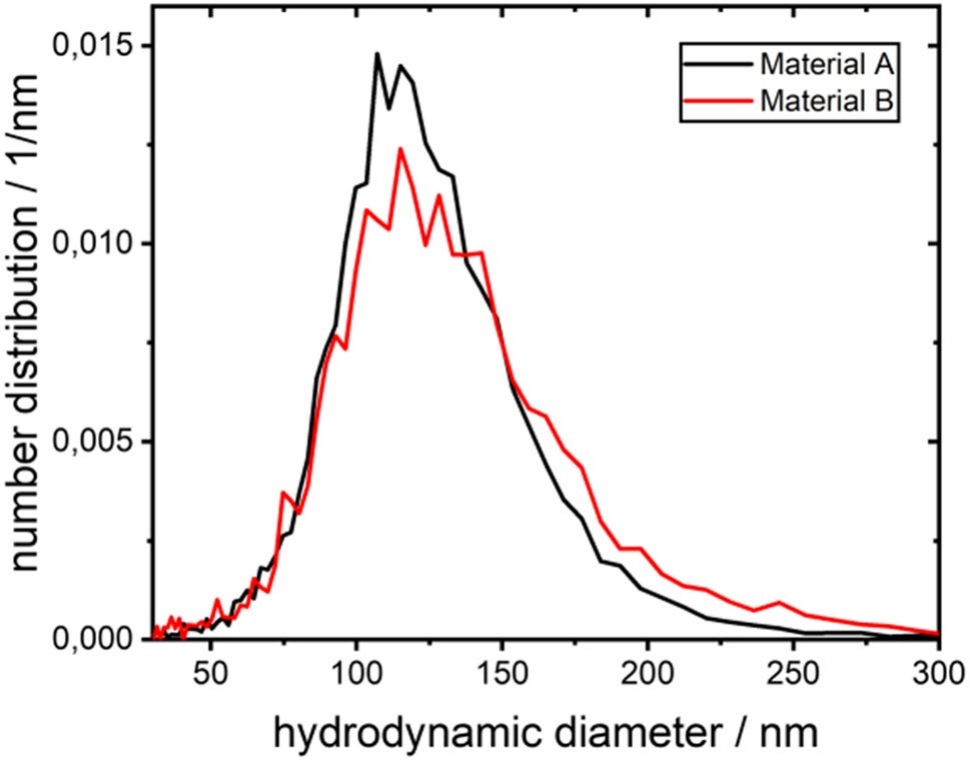
Hydrodynamic diameter distribution obtained from NTA for material A and B

NTA can be used to provide information on the diffusion coefficient of the analyte. The resulting number-based distributions for material A and B are depicted in Fig. 4. The median value of the hydrodynamic diameter distribution of material A (122 nm) is close to the theoretical hydrodynamic diameter of 120 nm calculated based on the electron-microscopy dimensions (175 × 70 nm) of wild-type VSV (David-West and Labzoffsky 1968) and a flat rod hydrodynamic model (Hansen 2004). Material B shows a slight shift to higher hydrodynamic diameters with a median hydrodynamic diameter of 129 nm.

From calculations (Hansen 2004) and simulations (Juba et al. 2017), the ratio of the hydrodynamic diameter of a dimer (side-by-side or head-to-head) and monomer rod can be approximated to be within the range of 1.3 to 1.4. Based on the given median value of Material A, dimeric rods could be expected to have a hydrodynamic diameter of approximately 160–175 nm, which could contribute to the slight tailing to higher diameters in Fig. 4.

Although, both NTA as well as AUC provide property distributions, namely sedimentation and diffusion coefficients, the simultaneous information on those quantities for the individual species is lost, as the provision of both parameters via AUC becomes increasingly difficult with high sedimentation coefficients, low diffusion coefficients and substantial dispersity of the investigated particles. Combining property distributions (e.g., $s,D, \overline{v }, M$) originating from different devices to provide multidimensional property distributions (e.g., composition vs. length vs. diameter) has great potential (Furat et al. 2020), but goes beyond current capabilities.

However, already with the results from NTA and AUC available, the molar mass of VSV-GP was estimated according to Eq. 4. Based on the median values of $s,D$ as well as the apparent partial specific volume associated with the median of the (D_2_O) cumulative sedimentation coefficient distributions in Fig. 3, the molar mass of material A can be given as $M=333 MDa$, while material B exhibits $M=315 MDa$, which is in line with the large range reported in the literature (Ware et al. 1973; Hartford et al. 1975; Thomas et al. 1985).

## Summary and conclusion

Within this study, AUC was used to characterize the oncolytic virus VSV-GP. Although, the fractionating method of AUC offers the opportunity to investigate dispersity in samples, limitations of the method occur due to the multidimensionality and complexity of enveloped virus particles. As the sedimentation coefficient depends on geometry, mass, and density, several different AUC experiments that aim at isolating individual disperse properties should be conducted. Especially, for large viral particles, other techniques that provide additional information, like nanoparticle tracking analysis, can help in providing more information on the quality of viral therapeutics. Overall, AUC can be a valuable tool to give information on disperse properties such as size, density, and mass, to support process and formulation development of viral therapeutics.

## Data Availability

The experimental data generated and analyzed during the present study are available from the corresponding author on reasonable request.
